# Bibliometric analysis and visualization of endocrine therapy for breast cancer research in the last two decade

**DOI:** 10.3389/fendo.2023.1287101

**Published:** 2023-12-05

**Authors:** Dasong Wang, Yan Yang, Lei Yang, Hongwei Yang

**Affiliations:** Department of Breast and Thyroid Surgery, Suining Central Hospital, Suining, Sichuan, China

**Keywords:** breast cancer, endocrine therapy, bibliometric analysis, biclustering analysis, visualization

## Abstract

**Background:**

Breast cancer endocrine therapy research has become a crucial domain in oncology since hormone receptor-positive breast cancers have been increasingly recognized, and targeted therapeutic interventions have been advancing over the past few years. This bibliometric analysis attempts to shed light on the trends, dynamics, and knowledge hotspots that have shaped the landscape of breast cancer endocrine therapy research between 2003 and 2022.

**Methods:**

In this study, we comprehensively reviewed the scientific literature spanning the above-mentioned period, which included publications accessible through the database of the Web of Science (WOS) and the National Center for Biotechnology Information (NCBI). Next, a systematic and data-driven analysis supported by sophisticated software tools was conducted, such that the core themes, prolific authors, influential journals, prominent countries, and critical citation patterns in the relevant research field can be clarified.

**Results:**

A continuous and substantial expansion of breast cancer endocrine therapy research was revealed over the evaluated period. A total of 1,317 scholarly articles were examined. The results of the analysis suggested that research on endocrine therapy for breast cancer has laid a solid basis for the treatment of hormone receptor-positive breast cancer. From a geographical perspective, the US, the UK, and China emerged as the most active contributors, illustrating the global impact of this study. Furthermore, our analysis delineated prominent research topics that have dominated the discourse in the past two decades, including drug therapy, therapeutic efficacy, molecular biomarkers, and hormonal receptor interactions.

**Conclusion:**

This comprehensive bibliometric analysis provides a panoramic view of the ever-evolving landscape of breast cancer endocrine therapy research. The findings highlight the trajectory of past developments while signifying an avenue of vast opportunities for future investigations and therapeutic advancements. As the field continues to burgeon, this analysis will provide valuable guidance for to researchers toward pertinent knowledge hotspots and emerging trends, which can expedite the discoveries in the realm of breast cancer endocrine therapy.

## 1 Introduction

Breast cancer stands as one of the most prevalent and life-threatening diseases affecting women worldwide (1). Its complex nature and the diverse range of factors contributing to its development have fueled extensive research efforts to uncover effective treatment strategies (2–4). Among these, endocrine therapy has emerged as a pivotal method, offering new hope to countless breast cancer patients (5, 6). This study focuses on the critical exploration of breast cancer endocrine therapy, delving into its mechanisms, the evolution of treatment strategies, and the remarkable impact it has had on improving patient results.

Breast cancer endocrine therapy has transformed from a relatively obscure treatment option to a cornerstone of breast cancer management over the past few decades (7–9), which has been fueled by the deepened understanding of the hormone receptor status in breast cancer cells (10, 11). Estrogen and progesterone receptors have been reported to play vital roles in tumor growth, which has underpinned targeted interventions (12, 13). Endocrine therapy, in essence, disrupts the hormonal signals that fuel tumor growth, effectively slowing down or even halting the progression of the disease (14–16).

This study endeavor seeks to provide a comprehensive overview of the multifaceted field of breast cancer endocrine therapy. It encompasses the historical development of this treatment modality, including milestones such as the discovery of tamoxifen and aromatase inhibitors (17, 18). The emergence of novel agents and combination therapies, along with the identification of predictive biomarkers (19), is reshaping the landscape of breast cancer treatment (20), such that new avenues are provided for personalized treatment. Its remarkable success in improving survival rates and enhancing the quality of life for patients underscores its significance (21–23). In this study, the progress made was demonstrated, and a basis is laid for other researchers to explore the intricacies of this field further.

Bibliometrics refers to a quantitative method used for the analysis and assessment of scientific literature, utilizing statistical methods and visualization techniques (24). This discipline facilitates the discovery of research focal points and emerging trends in a specific domain, offering valuable perspectives into the present status of the field and steering forthcoming research endeavors. As an illustration, through bibliometric examination, scholars have effectively evaluated the ongoing research landscape pertaining to subjects like microneedles and chronic wounds (25, 26). By providing an objective portrayal of research patterns, bibliometrics plays a crucial role in shaping and directing future research directions.

The goal of this investigation is to perform a thorough assessment of the current body of literature related to breast cancer endocrine therapy using a bibliometric analysis method. The intention is to enhance comprehension of the breast cancer endocrine therapy research domain by employing co-word biclustering analysis. The anticipated results of this study are poised to assist researchers in achieving a more effective and precise insight into the trends and advancements in breast cancer endocrine therapy research.

## 2 Methods

### 2.1 Data source and search methods

**Figure 1:**
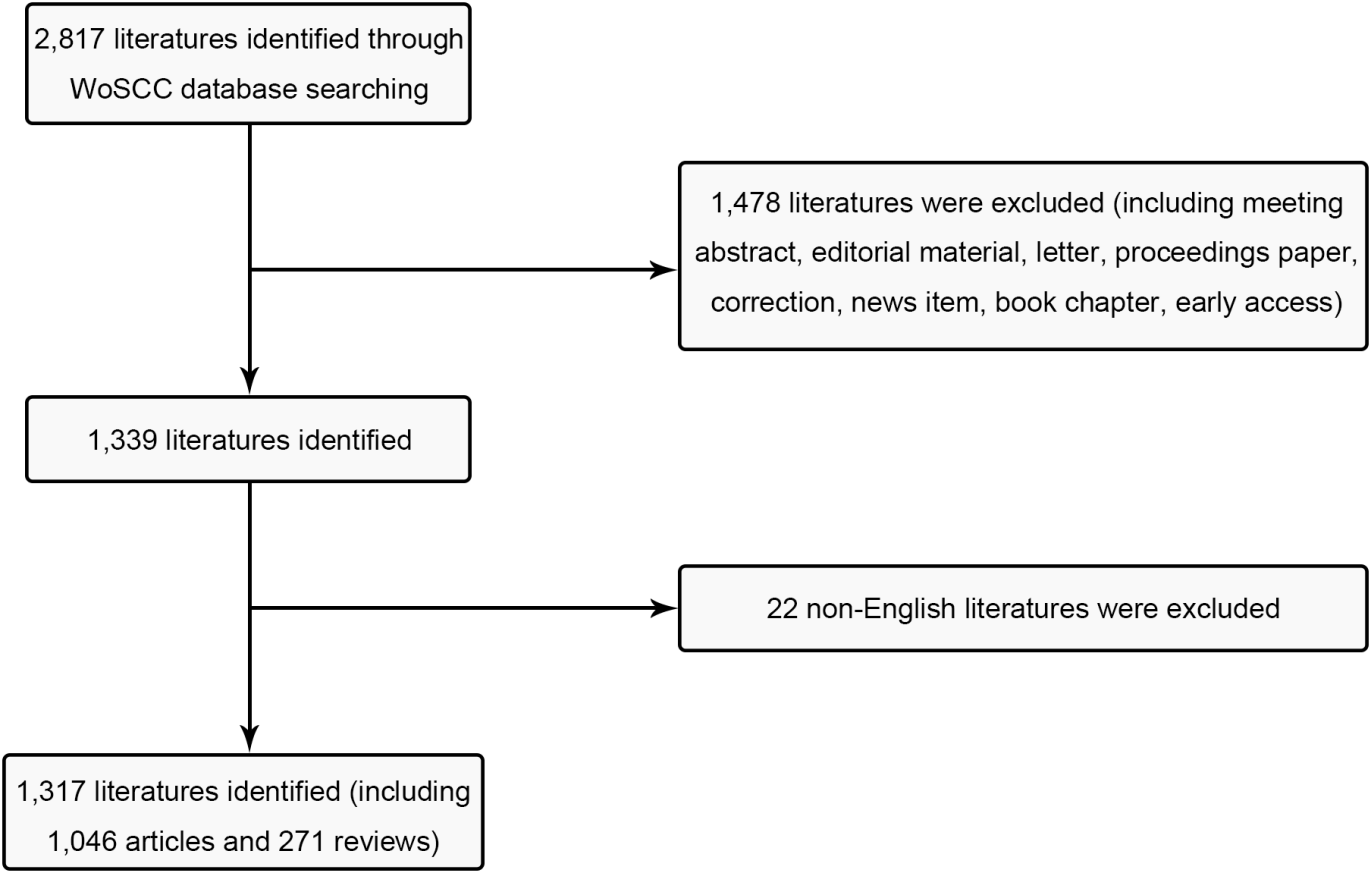
Flow chart of inclusion and exclusion.

The Science Citation Index Extension (SCI-E) in WOS was employed in accordance with the search parameters as follows: TI = (Breast cancer) and (Endocrine therapy) and language = English, covering the time span from Jan. 1, 2003, to Dec. 31, 2022. 2817 articles were gathered, and subsequently, specific types of literature were excluded, encompassing (1) early access; (2) book chapter; (3) news item (4) correction; (5) proceedings paper; (6) letter; (7) editorial material; (8) meeting abstract (**Figure 1**). The function presented by the NCBI for the retrieval of Medical Subject Heading (MeSH) terms was adopted for co-word clustering analysis. The analysis timeframe was set from 2003 to 2022, with “Breast cancer endocrine therapy” designated as the MeSH term. We retrieved all publications on July 10, 2023 for mitigating any potential biases caused by database updates.

### 2.2 Data collection

The search results were independently evaluated by two reviewers, which included aspects (e.g., H-index, citation count, geographical location, author details, publication date, and title). The reviewers achieved a substantial overall agreement ratio of 0.90. In instances where discrepancies in evaluations arose, a third reviewer was engaged to adjudicate on the inclusion of the respective data.

The data utilized for analysis was imported into two different platforms: the online analysis platform of bibliometrics (http://bibliometric.com/) and VOSviewer V1.6.17 (Leiden University, Leiden, the Netherlands), both in the “Tab Delimited File” format. In the case of CiteSpace V 6.2.R4 (Drexel University, Philadelphia, PA, United States), the data was imported in the “Plain Text File” format. Additionally, MeSH terms obtained from NCBI were initially formatted as “PubMed” and subsequently input into BICOMB V2.02 for analysis. Following this, we used gCLUTO V1.0 (Graphical Clustering Toolkit) to carry out biclustering visualization based on the co-word matrix file produced through the investigation.

## 3 Data investigation

### 3.1 Bibliometric analysis and geographical distribution

We visualized international collaborations and contributions at national/regional levels based on the Bibliometrics online analysis platform. VOSviewer facilitated the creation of a vivid clustering visualization, with a primary focus on assessing institutions’ inter-agency cooperation. Furthermore, we adopted the number of journals published as the standard for the density clustering visualization. In our utilization of VOSviewer, we opted for the “bibliographic data-based map creation” data format and selected “Co-authorship” as the analysis category, such that we generated the “Density Visualization” graphic. Additionally, we harnessed CiteSpace for forecasting subsequent research directions and identifying chart research trends and burst words dependent on time. When data import into CiteSpace was completed, the “Remove duplicates” function should be employed prior to the “keyword” configuration as node types. To be specific, we set “the number of states” at 2. The 2023 Journal Citation Report (JCR) was adopted for determining journal impact factors (IF).

### 3.2 Co-word biclustering analysis

BICOMB and gCLUTO were employed to conduct biclustering analysis on significant MeSH terms and MeSH subheadings, with the objective of identifying research focal points. In BICOMB, a file in “PubMed-2” format was generated, and the relevant MeSH term data was imported. The “Extract” function was used to configure “main topic + sub-topic”. Following this step, the primary MeSH terms were transformed into a matrix using specialized software, resulting in a co-word matrix that highlighted high-frequency MeSH terms. Subsequently, this co-word matrix was introduced into gCLUTO, where parameters such as the “Number of Clusters” were defined, and the “Cluster Method” function was set to “Repeated Bisection”. Finally, the mountain visualization techniques were employed to present the results of the biclustering analysis. This biclustering analysis offers valuable insights into the research hotspots in the field of breast cancer endocrine therapy.

## 4 Results

### 4.1 Investigation of publications output

**Figure 2:**
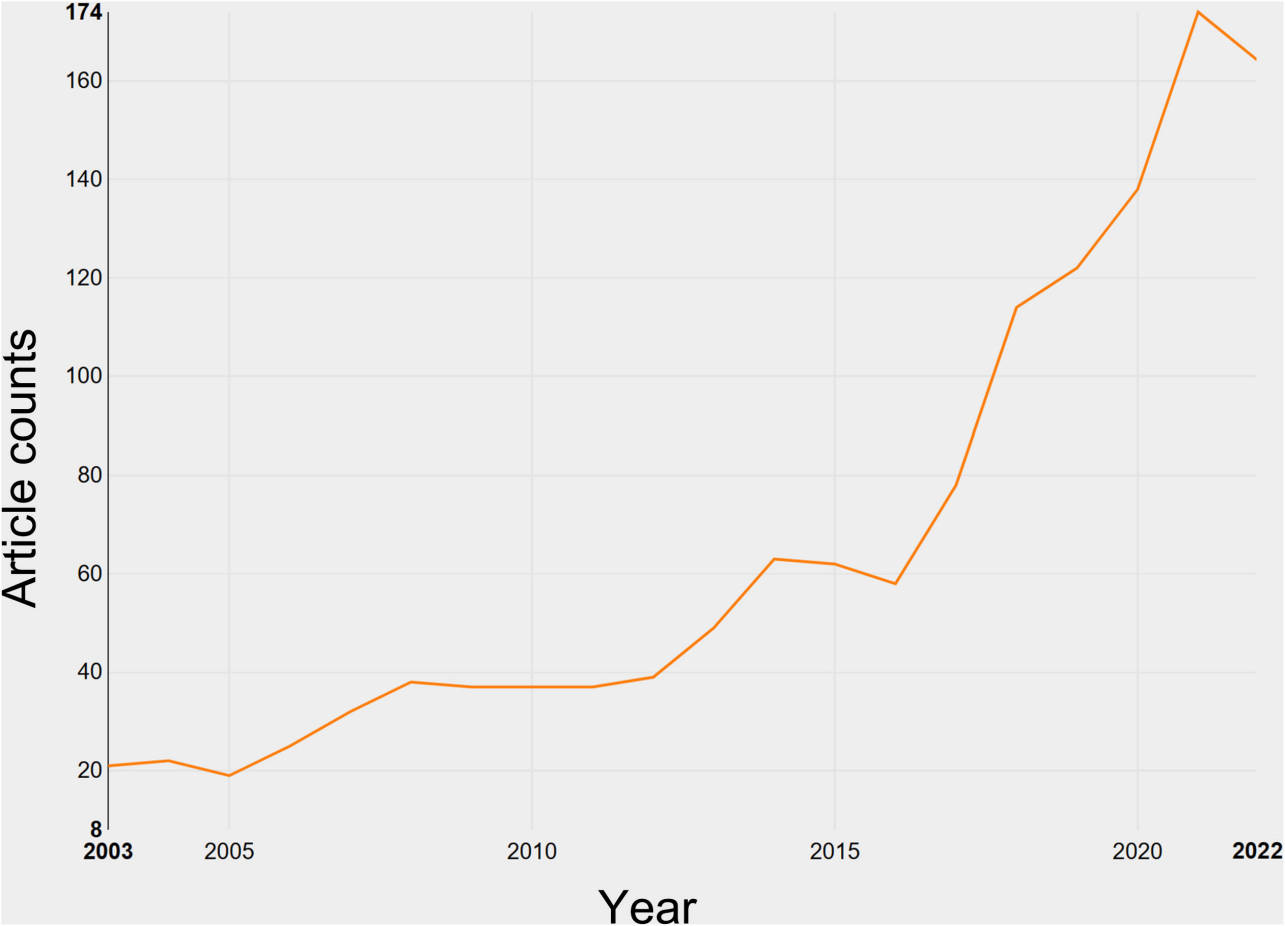
Growth of publications on breast cancer endocrine therapy (2003–2022).

A total of 1,046 research articles and 271 review articles, all in accordance with the inclusion criteria, were identified in the realm of breast cancer endocrine therapy (see **Figure 1**). An examination of the publication trends from 2003 to 2022 reveals a continuous growth pattern, particularly notable in 2016 (see **Figure 2**). It’s worth noting that, as of the current date, 174 articles have already been published in 2022, which signifies more than an eightfold increase compared to the number published in 2003.

### 4.2 The contributions of nations and institutions to global publications

**Figure 3:**
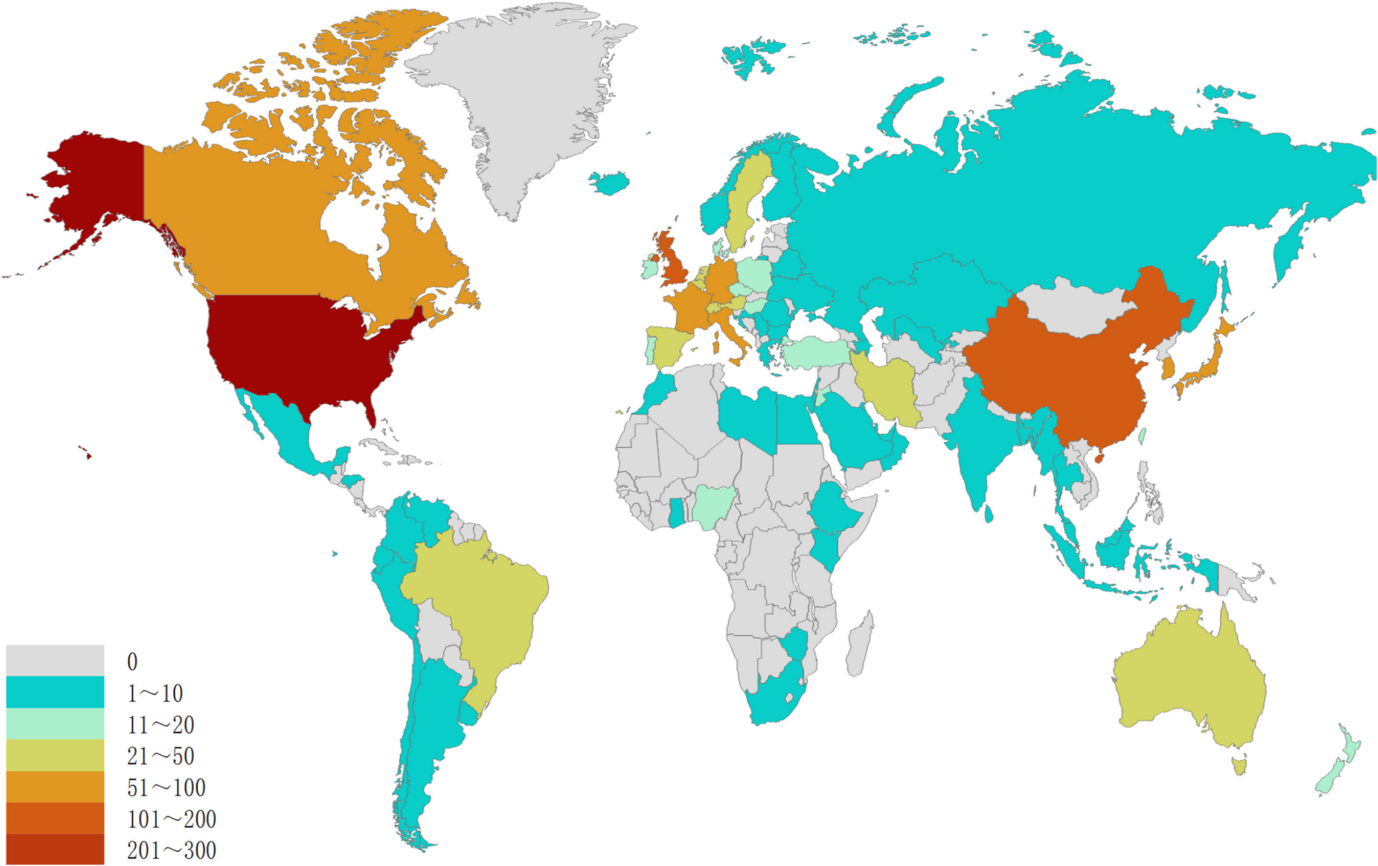
Geographical distribution of retrieved articles in breast cancer endocrine therapy (2003–2022).

**Figure 4:**
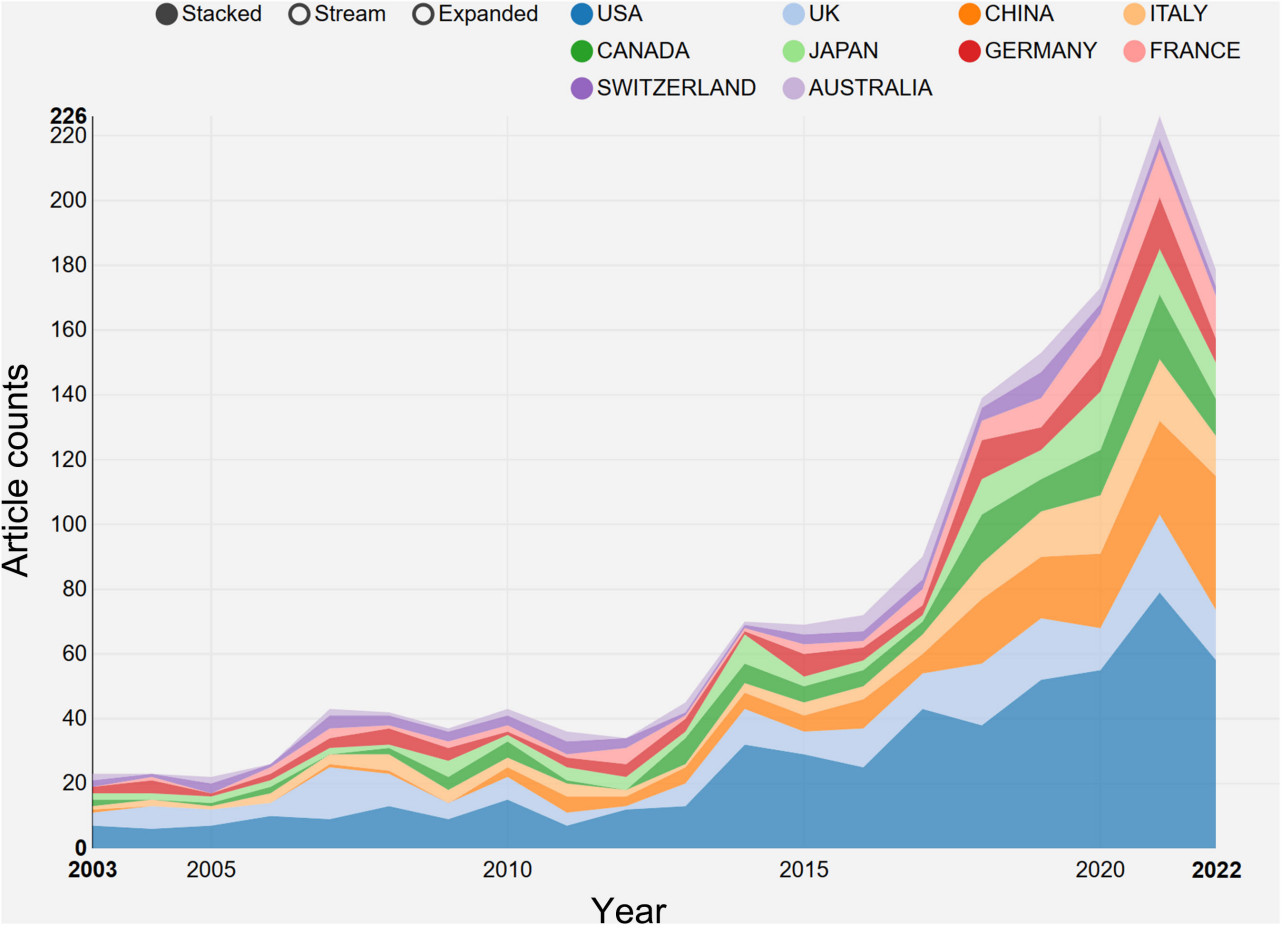
The growth trends of the top 10 nations/regions in breast cancer endocrine therapy (2003–2022).

**Figure 5:**
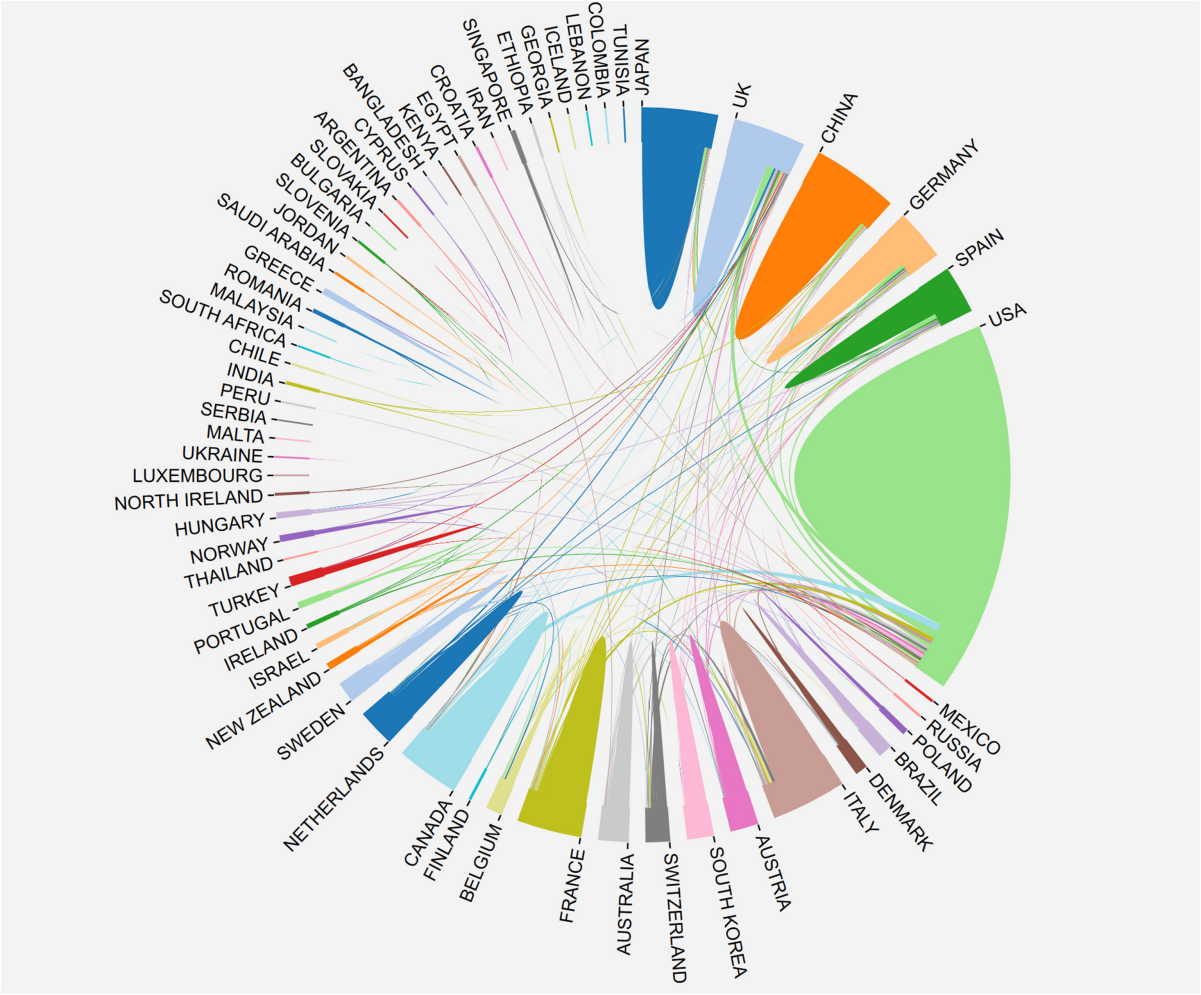
The network map of country/region’ cooperation.

**Figure 6:**
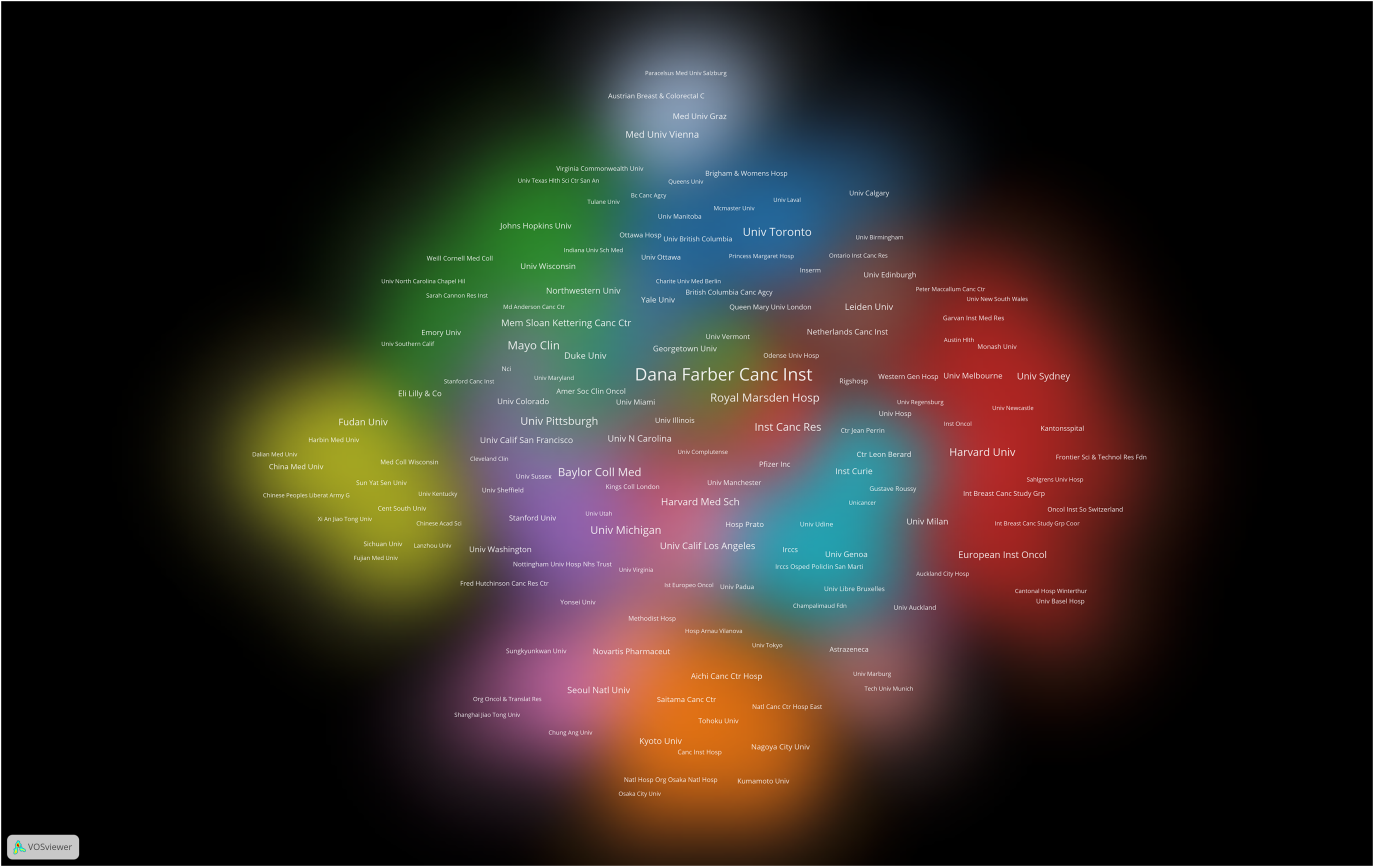
Cluster density visualization map of institutions on breast cancer endocrine therapy (2003–2022).

The research on breast cancer endocrine therapy originates from a diverse set of 68 countries and regions. After importing the data, a thermal world map (**Figure 3**) illustrates that the above-mentioned articles are predominantly concentrated in East Asia, North America, and Western Europe. Notably, the US (n=525) emerged as the leading contributor, closely followed by England (n=176) and China (n=173) (**Table 1**). The publication growth trajectories for major countries are displayed in **Figure 4**. As indicated by an investigation into national and regional collaborations, the US and England engaged in the most frequent cooperation (**Figure 5**). Centrality refers to a vital indicator of whether a country is involved in collaboration worldwide, indicating a more significant effect with higher centrality values. The above-mentioned results indicated that the US exhibited the most prominent centrality (center = 0.26), followed by Canada (0.18) and England (0.15). Among research institutions, the top five include Harvard University (n=115), Dana Farber Cancer Institute (n=82), University of Texas System (n=82), Unicancer (n=65), as well as University of California System (n=58) (**Table 1**). Using VOSviewer, we investigated inter-agency collaborations in accordance with co-authorship while visualizing the above-described collaborations as density clusters (**Figure 6**). The above-mentioned inter-agency partnership examination suggested that ten different clusters constituted the complete agencies, each of which was indicated by a unique color.

**Table 1:** Top ten productive Country/region and institutions on breast cancer endocrine therapy (2003–2022).

Rank	Country/region	Article counts	Centrality	Total number of citations	Average number of citations	Institutions	Article counts	Total number of citations
1	USA	525	0.26	23,008	43.82	Harvard University	115	7,905
2	England	176	0.15	10,747	61.06	Dana Farber Cancer Institute	82	6,047
3	China	173	0.11	3,391	19.6	University of Texas System	82	5,845
4	Italy	120	0.05	7,141	59.51	Unicancer	65	3,503
5	Canada	115	0.18	1,403	17.32	University of California System	58	4,446
6	Japan	109	0.08	4,734	43.43	Royal Marsden Nhs Foundation Trust	57	7,417
7	Germany	92	0.13	8,385	91.14	Utmd Anderson Cancer Center	57	5,071
8	France	79	0.06	4,859	61.51	University of London	50	4,527
9	Switzerland	59	0.06	4,394	74.47	Udice French Research Universities	47	1,404
10	Australia	58	0.10	5,358	92.38	Mayo Clinic	40	2,498

### 4.3 Journals publishing research on breast cancer endocrine therapy

**Figure 7:**
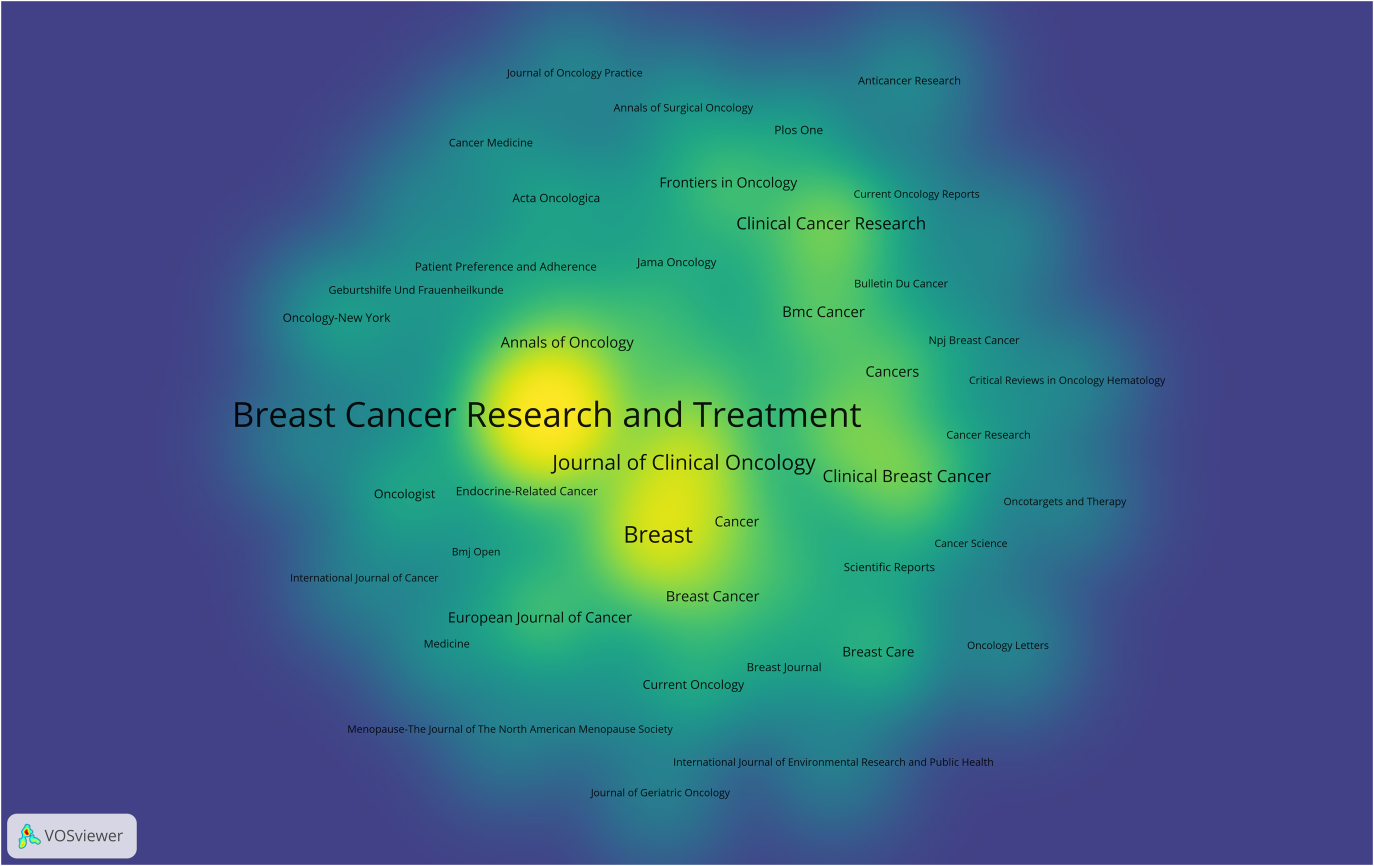
Density visualization map of journals on breast cancer endocrine therapy (2003–2022).

A cumulative total of 309 academic journals have made significant contributions to the dissemination of research concerning breast cancer endocrine therapy. **Figure 7** provides an overview of the journals that have particularly excelled in this study domain. Among the 1,317 articles dedicated to breast cancer endocrine therapy, 419 of them (constituting 31.81%) found their place in the top 10 journals (**Table 2**). Notably, the leading position in terms of study publications was secured by Breast Cancer Research and Treatment, followed by Breast and Drug Development and Journal of Clinical Oncology, collectively accounting for 18.07% of all articles in this area. In the category of journals that have published more than 10 articles, it’s noteworthy that Annals of Oncology (IF: 50.5) emerged as the frontrunner, followed by Annals of Oncology (IF: 45.3) and Cancer Treatment Reviews (IF: 11.8). All three of the above-mentioned journals hold the prestigious Q1 classification in line with the JCR 2023 standard.

**Table 2:** Top ten journals in the publication on breast cancer endocrine therapy (2003–2022).

Rank	Journal	H-index	IF (2023)	Article counts	Percentage (N=1,317)	Total number of citations	Average number of citations
1	Breast Cancer Research and Treatment	31	3.8	124	9.415%	2,849	22.98
2	Breast	19	3.9	64	4.860%	990	15.47
3	Journal of Clinical Oncology	37	45.3	50	3.797%	7,887	157.74
4	Clinical Cancer Research	21	11.5	33	2.506%	1,861	56.39
5	Clinical Breast Cancer	10	3.1	32	2.430%	383	11.97
6	Breast Cancer Research	9	7.4	27	2.050%	1,072	39.7
7	Annals of Oncology	9	50.5	23	1.746%	1,657	72.04
8	Bmc Cancer	9	3.8	23	1.746%	459	19.96
9	Cancers	8	5.2	22	1.670%	258	11.73
10	Breast Cancer	7	4.0	21	1.595%	224	10.67

### 4.4 Authors’ contributions to breast cancer endocrine therapy research

A total of 6,094 distinct authors made active contribution to this investigation, with the top 10 most prolific researchers listed in **Table 3**. Colleoni, M. from IRCCS European Institute of Oncology, Hannover, Germany. Ellis, M. J. from Baylor College of Medicine, Baylor University, State of Texas, USA. Goldhirsch, A. from IRCCS European Institute of Oncology, Hannover, Germany. We analyzed pinpoint articles with a significant citation frequency, and we present the top 10 articles in the breast cancer endocrine therapy field. “Fulvestrant plus palbociclib versus fulvestrant plus placebo for treatment of hormone-receptor-positive, HER2-negative metastatic breast cancer that progressed on previous endocrine therapy (PALOMA-3): final analysis of the multicenter, double-blind, phase 3 randomized controlled trial” published by Cristofanilli, M. et al. in LANCET ONCOLOGY in 2016 (n=1,079) was the most frequently cited study (**Table 4**).

**Table 3:** The top ten most productive authors contributed to publications in breast cancer endocrine therapy research (2003–2022).

Rank	Author	Article counts	H-index	Total number of citations	Average number of citations
1	Colleoni M	29	15	3,855	132.93
2	Ellis MJ	26	17	1,750	67.31
3	Goldhirsch A	21	14	1,853	88.24
4	Harbeck N	20	14	3,071	153.55
5	Toi M	19	11	1,900	100
6	Gnant M	18	13	2,672	148.44
7	Burstein HJ	17	15	2,892	170.12
8	Davidson NE	17	12	2,218	130.47
9	Iwata H	17	9	1,346	79.18
10	Masuda N	17	10	2,617	153.94

**Table 4:** Top ten cited articles on breast cancer endocrine therapy (2003–2022).

Rank	Title	Journal	Corresponding author	Publication year	Total citations
1	Fulvestrant plus palbociclib versus fulvestrant plus placebo for treatment of hormone-receptor-positive, HER2-negative metastatic breast cancer that progressed on previous endocrine therapy (PALOMA-3): final analysis of the multicentre, double-blind, phase 3 randomised controlled trial	LANCET ONCOLOGY	Cristofanilli, M	2016	1,079
2	MONARCH 2: Abemaciclib in Combination With Fulvestrant in Women With HR+/HER2-Advanced Breast Cancer Who Had Progressed While Receiving Endocrine Therapy	JOURNAL OF CLINICAL ONCOLOGY	Sledge, GW	2017	862
3	Endocrine Therapy plus Zoledronic Acid in Premenopausal Breast Cancer	NEW ENGLAND JOURNAL OF MEDICINE	Gnant, M	2009	842
4	20-Year Risks of Breast-Cancer Recurrence after Stopping Endocrine Therapy at 5 Years	NEW ENGLAND JOURNAL OF MEDICINE	Pan, HC	2017	799
5	Five years of letrozole compared with tamoxifen as initial adjuvant therapy for postmenopausal women with endocrine-responsive early breast cancer: Update of study BIG 1-98	JOURNAL OF CLINICAL ONCOLOGY	Coates, A. S	2007	732
6	American Society of Clinical Oncology Clinical Practice Guideline: Update on Adjuvant Endocrine Therapy for Women With Hormone Receptor-Positive Breast Cancer	JOURNAL OF CLINICAL ONCOLOGY	Burstein, HJ	2010	574
7	Adjuvant Endocrine Therapy for Women With Hormone Receptor-Positive Breast Cancer: American Society of Clinical Oncology Clinical Practice Guideline Focused Update	JOURNAL OF CLINICAL ONCOLOGY	Burstein, HJ	2014	540
8	Progesterone receptor status significantly improves outcome prediction over estrogen receptor status alone for adjuvant endocrine therapy in two large breast cancer databases	JOURNAL OF CLINICAL ONCOLOGY	Bardou, VJ	2003	540
9	FGFR1 Amplification Drives Endocrine Therapy Resistance and Is a Therapeutic Target in Breast Cancer	CANCER RESEARCH	Turner, N	2010	526
10	Overall Survival with Ribociclib plus Endocrine Therapy in Breast Cancer	NEW ENGLAND JOURNAL OF MEDICINE	Im, SA	2019	493

### 4.5 Research hotspots of breast cancer endocrine therapy

**Figure 8:**
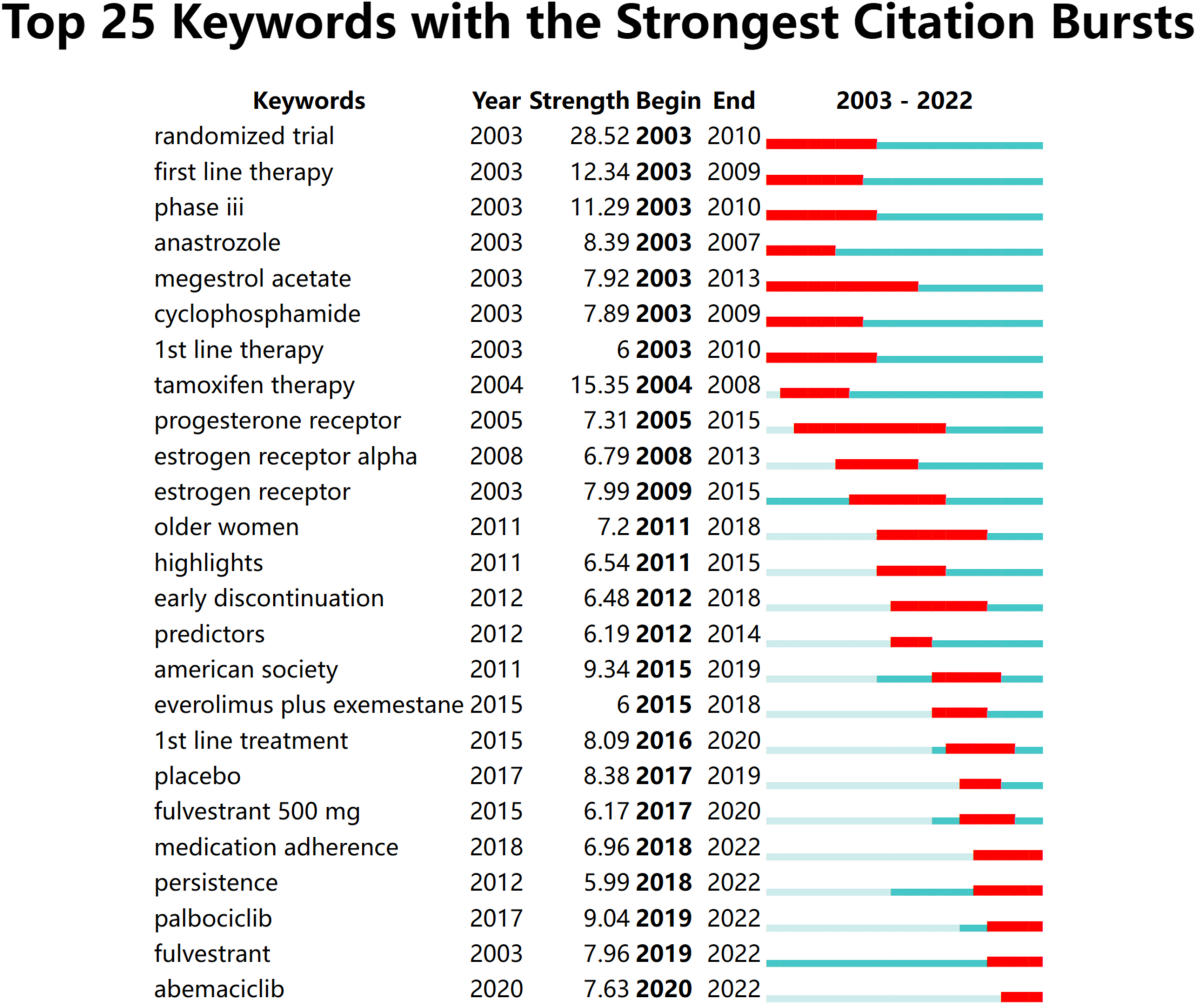
The top 25 burst words from 2003 to 2022.

**Figure 9:**
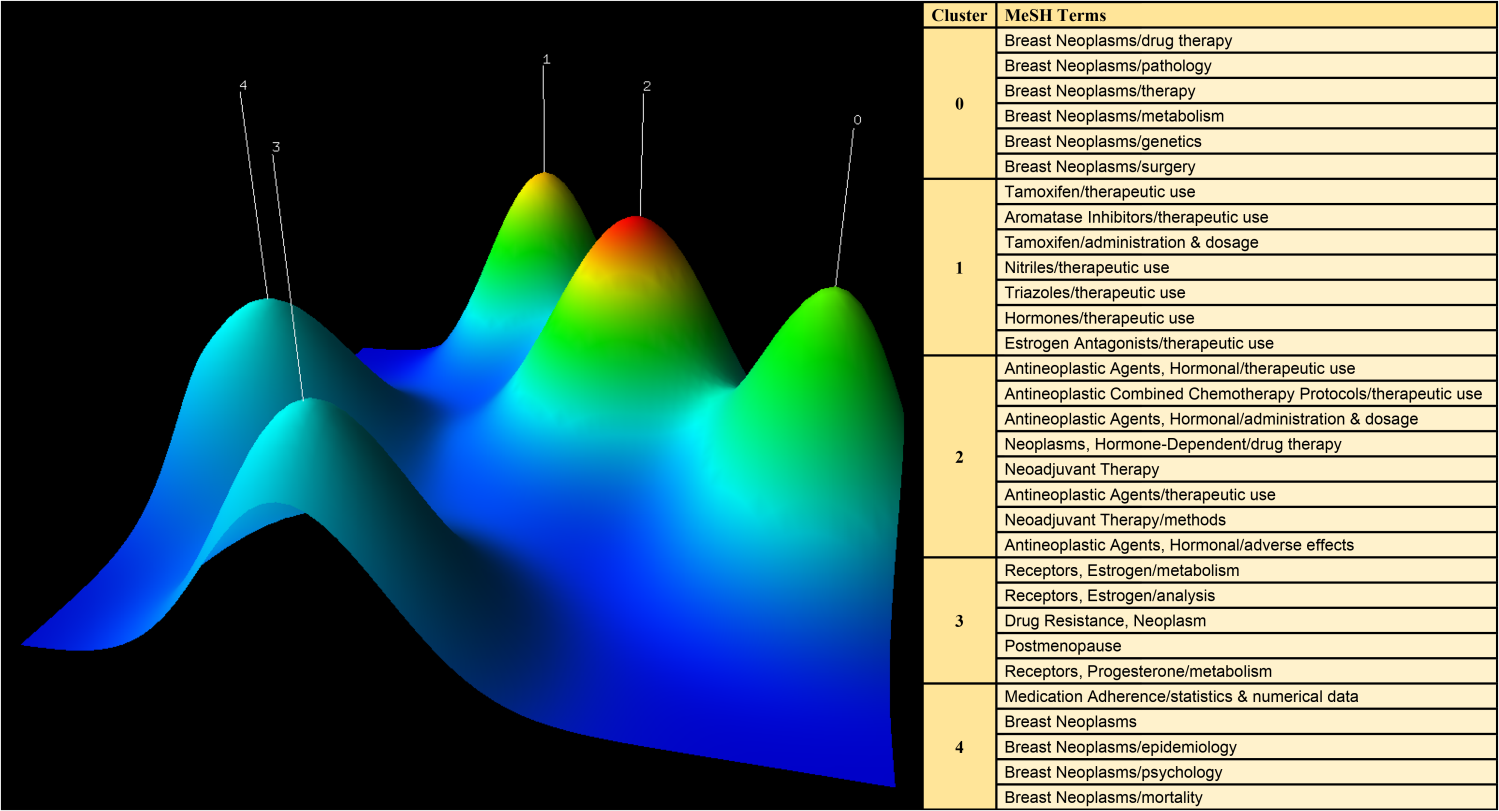
Mountain visualization of major MeSH terms/MeSH subheading terms of articles on breast cancer endocrine therapy.

CiteSpace was employed to extract keywords from a dataset consisting of 5,220 literature sources. This process resulted in the identification of the top 25 burst words spanning the timeframe from 2003 to 2022. The above-described dynamic burst words serve to illuminate the evolving trends in research hotspots (**Figure 8**). Furthermore, a total of 1,326 significant MeSH terms/MeSH subheadings were discovered, with an accumulated frequency of 6,279 instances. According to the G-index criteria, high-frequency terms were defined as those recurring more than 31 times (**Table 5**). To visualize the above-mentioned research hotspots, biclustering analysis was executed using BICOMB and gCLUTO. BICOMB facilitated the creation of a co-word matrix, which was subsequently imported into gCLUTO for mountain graph (**Figure 9**) that revealed five distinctive clusters in the research field. The gap between the above-described mountains denotes the degree of correlation between clusters, with mountain height and volume signifying internal similarity and term coverage. Furthermore, the color transitions from red to green on the peak reflect the standard deviation, indicating deviation levels. The biclustering analysis of publications yielded five different clusters: (I) The overall treatment of breast cancer; (II) Hormonal treatments of breast cancer; (III) Neoadjuvant therapy of breast cancer; (IV) Estrogen receptors of breast cancer; (V) Adherence and survival rates of breast cancer.

**Table 5:** Major MeSH terms/MeSH subheadings from the included publications on breast cancer endocrine therapy (n=1,326).

Rank	Major MeSH terms/MeSH subheadings	Frequency	Proportion of frequency (%)	Cumulative percentage (%)
1	Breast Neoplasms/drug therapy	1165	18.5539	18.5539
2	Antineoplastic Agents, Hormonal/therapeutic use	476	7.5808	26.1347
3	Tamoxifen/therapeutic use	180	2.8667	29.0014
4	Breast Neoplasms/pathology	149	2.3730	31.3744
5	Aromatase Inhibitors/therapeutic use	144	2.2934	33.6678
6	Antineoplastic Combined Chemotherapy Protocols/therapeutic use	142	2.2615	35.9293
7	Breast Neoplasms/therapy	127	2.0226	37.9519
8	Breast Neoplasms/metabolism	95	1.5130	39.4649
9	Breast Neoplasms/genetics	94	1.4971	40.9619
10	Receptors, Estrogen/metabolism	82	1.3059	42.2679
11	Antineoplastic Agents, Hormonal/administration & dosage	75	1.1945	43.4623
12	Neoplasms, Hormone-Dependent/drug therapy	63	1.0033	44.4657
13	Tamoxifen/administration & dosage	52	0.8282	45.2938
14	Receptors, Estrogen/analysis	48	0.7645	46.0583
15	Nitriles/therapeutic use	46	0.7326	46.7909
16	Triazoles/therapeutic use	46	0.7326	47.5235
17	Neoadjuvant Therapy	44	0.7007	48.2242
18	Antineoplastic Agents, Hormonal/adverse effects	43	0.6848	48.9091
19	Breast Neoplasms/surgery	43	0.6848	49.5939
20	Breast Neoplasms/psychology	42	0.6689	50.2628
21	Estrogen Antagonists/therapeutic use	41	0.6530	50.9158
22	Antineoplastic Agents/therapeutic use	41	0.6530	51.5687
23	Breast Neoplasms/epidemiology	38	0.6052	52.1739
24	Drug Resistance, Neoplasm	37	0.5893	52.7632
25	Breast Neoplasms	35	0.5574	53.3206
26	Postmenopause	35	0.5574	53.8780
27	Medication Adherence/statistics & numerical data	34	0.5415	54.4195
28	Neoadjuvant Therapy/methods	34	0.5415	54.9610
29	Receptors, Progesterone/metabolism	32	0.5096	55.4706
30	Breast Neoplasms/mortality	31	0.4937	55.9643
31	Hormones/therapeutic use	31	0.4937	56.4580

## 5 Discussion

The results of the bibliometric analysis highlight a substantial growth in the body of literature concerning breast cancer endocrine therapy between 2003 and 2022. This expansion has rendered it increasingly challenging to stay abreast of the prevailing research focal points. To address the above-mentioned challenge, we conducted a systematic examination by employing both bibliometric and biclustering analysis methodologies. This comprehensive study involved the retrieval of pertinent articles from the WOS and NCBI databases, encompassing a two-decade timeframe. The findings of this investigation are anticipated to furnish valuable perspectives on research hotspots and offer insights into future trends.

### 5.1 Overview of breast cancer endocrine therapy research

This investigation was aimed at evaluating the national academic contributions and the quality of research in the field of breast cancer endocrine therapy. The above evaluation was based on a wide variety of factors, which comprised the quantity of articles, the total number of citations, centrality, and the average number of citations generated by each country or region. Notably, the findings reveal that the US holds the most significant influence in the realm of breast cancer endocrine therapy research, amassing a total of 23,008 citations. Following closely, England and Germany garnered 10,747 and 8,385 citations, respectively. Noteworthily, breast cancer endocrine therapy research in England displayed a declining trend. In contrast, China has maintained a consistent increase in the number of breast cancer endocrine therapy publications, surpassing the US in 20220. Despite this, China’s impact remains somewhat modest (centrality=0.11), ranking fifth among the top 10 countries. Furthermore, the quality of breast cancer endocrine therapy publications from China can be further improved, since the average number of citations per study is relatively low (19.6) and ranks ninth among the top 10 countries. The thermal world map underscores regions that are investing more significantly in breast cancer endocrine therapy research. The results suggest that some regions (e.g., Africa, South and Central Asia, and Eastern Europe) have limited involvement in breast cancer endocrine therapy research and may contribute more in this field by leveraging the benefit from international collaboration and support.

The findings of this analysis reveal that a majority of the top 10 institutions contributing to the field of breast cancer endocrine therapy are affiliated with the US (n=7) and England (n=2), highlighting their substantial impact. We used a vivid density visualization technique to illustrate the different clusters of institutions, allowing for an intuitive assessment of their collaborative relationships. Institutions engaged in close collaboration are depicted in the same colored cloud, and the size of the cluster area corresponds to the group’s significance in the field. Furthermore, the size of each institution’s name reflects the extent of its cooperation with other institutions. Harvard University, Dana Farber Cancer Institute, University of Texas System, and University of California System have been identified as prominent contributors to research in breast cancer endocrine therapy.

The foremost journals in the field of breast cancer endocrine therapy (e.g., the University of California System, Breast, and Journal of Clinical Oncology) have jointly made a significant contribution (constituting more than 18%) to the body of work in this field. Breast Cancer Research and Treatment has firmly established itself as the preeminent journal in this domain, evident from its notably higher number of publications in the field of breast cancer endocrine therapy compared to its closest competitor. Furthermore, this journal holds the second-highest H-index among the top 10 journals, further affirming its pivotal role in advancing knowledge in the realm of breast cancer endocrine therapy. The above-mentioned observations underscore the influential contributions made by Breast Cancer Research and Treatment in shaping the discourse and progress of breast cancer endocrine therapy.

The analysis of burst words in this field demonstrated a shift in the primary research focus during the period from 2012 to 2014. The initial terms revolve around specific therapies, receptors, and predictors in articles involving older women. These articles shed light on the traditional approach that emphasized understanding the molecular subtypes of breast cancer, such as ER-positive and HER2-negative, and tailoring treatment regimens accordingly. During this phase, the scientific community sought to elucidate the efficacy of well-established endocrine therapies like tamoxifen and aromatase inhibitors. These findings were instrumental in shaping the early landscape of breast cancer endocrine therapy, which at the time was focused primarily on optimizing established therapies for specific patient groups. The latter terms suggest a more recent emphasis on treatment adherence, guidelines, and collaborative efforts. The focus has expanded beyond purely therapeutic aspects to encompass a holistic patient-centric approach. Researchers and clinicians now recognize the importance of ensuring that patients not only receive effective therapies but also adhere to their personalized prescribing regimens. This transition highlights an evolving research landscape, it reflects the broader adoption of precision medicine and patient-centric methods in clinical research and practice. The integration of cutting-edge genomics, transcriptomics, and proteomics has allowed for a more personalized approach to treatment. Biomarkers beyond ER and PR status, such as ESR1 and PIK3CA mutations, are being explored to better guide therapy selection. This evolution in research and practice signifies a promising trajectory towards improved treatment outcomes and enhanced patient well-being.

### 5.2 Five clustering hotspots of breast cancer endocrine therapy research.

#### 5.2.1 The overall treatment of breast cancer

Cluster 0 presents a correlation with the overall treatment of breast cancer. Breast cancer therapy research has witnessed significant advancements in the realm of drug design and development for the treatment of breast neoplasms. The exploration of novel drug therapies has been driven by a deep understanding of breast cancer’s underlying pathology, therapy landscape, and the diverse genetic and metabolic characteristics that govern the disease. The above-described efforts have yielded promising therapeutic interventions that hold potential for improved patient results.

The success of drug therapy for breast neoplasms is intrinsically linked to the detailed understanding of the disease’s pathology (27, 28). Researchers have identified key pathways and signaling cascades that drive tumor growth and progression by dissecting the intricacies of breast cancer at the cellular and molecular levels (29–31). The above identification has laid a basis for the rational design of targeted therapies that selectively disrupt the above-described pathogenic processes while minimizing collateral damage to healthy tissues. Such precision medicine methods are transforming breast cancer therapy by offering tailored treatments that address the specific biological characteristics of each patient’s tumor (32, 33).

Metabolic reprogramming in breast neoplasms acts as a promising avenue for therapeutic intervention (34–36). Aberrant metabolic pathways in cancer cells (e.g., increased glycolysis and altered lipid metabolism) offer potential targets for drug design (37–40). Compounds that selectively inhibit the above-mentioned metabolic adaptations have shown promise in preclinical articles and clinical trials, providing hope for more effective and less toxic treatment options. Furthermore, understanding the genetic underpinnings of breast cancer has allowed for the development of targeted therapies that exploit vulnerabilities in cancer cells driven by specific genetic mutations (41, 42), which further enriches the available treatment methods.

Drug therapy research has played a complementary role under the context of breast cancer surgery. Neoadjuvant therapies are increasingly being adopted to shrink tumors before surgical resection, which can increase the feasibility of breast-conserving surgeries and reduce the extent of mastectomies (43, 44). This integrated method underscores the significance of combining surgery with drug therapy to optimize patient results and underscores the evolving landscape of breast cancer therapy research.

#### 5.2.2 Hormonal treatments of breast cancer

Cluster 1 indicates the correlations with hormonal treatments of breast cancer. Breast cancer therapy research has made the breakthrough in using hormonal treatments for treating hormone receptor-positive breast cancers. Tamoxifen, a well-established selective estrogen receptor modulator, has been reported as a critical component of breast cancer treatment. Its therapeutic use spans early and advanced breast cancer cases, which can validate its versatility. The therapeutic effect of tamoxifen administered at precise dosages and following rigorous schedules is achieved by competitively binding to estrogen receptors, which can effectively block the proliferative signals of estrogen (45). The administration and dosage of Tamoxifen demand careful consideration since individual patient characteristics and tumor profiles can affect treatment response (46). Tamoxifen, a cornerstone in breast cancer therapy, underscores the significance of tailored hormonal treatments in improving patient results.

Aromatase Inhibitors have revolutionized the landscape of hormonal therapy for postmenopausal women with hormone receptor-positive breast cancer. The above-described agents, by inhibiting the conversion of androgens to estrogens, effectively lower systemic estrogen levels, thereby curtailing estrogen’s role in driving tumor growth (47, 48). Aromatase Inhibitors have demonstrated superiority over Tamoxifen in specific clinical scenarios, offering improved disease-free survival and reduced risk of recurrence (49, 50). As the above-mentioned agents continue to evolve, understanding their benefits, potential side effects, and optimal administration regimens is essential to guide clinical decisions and ensure the best results for patients.

In addition to Tamoxifen and Aromatase Inhibitors, a broader spectrum of hormonal therapies, including Nitriles, Triazoles, and other estrogen antagonists, has emerged as a promising avenue in breast cancer treatment (51, 52). The above-described agents offer diversified mechanisms of action, targeting various pathways involved in estrogen signaling. The therapeutic use of the above-mentioned agents has been investigated extensively, both individually and in combination with other treatments (53), to further optimize breast cancer therapy. As we delve deeper into the complexities of breast cancer biology, the above-described hormonal therapies hold the potential to provide more tailored and effective treatment options for a broader range of patients, emphasizing the evolving landscape of breast cancer therapy research.

#### 5.2.3 Neoadjuvant therapy of breast cancer

Cluster 2 presents the correlations with neoadjuvant therapy of breast cancer. Breast cancer therapy research has been pronouncedly enriched through the development and refinement of antineoplastic agents, particularly hormonal therapies. The above-mentioned agents are meticulously administered based on patient-specific factors (e.g., tumor type, hormone receptor status, and individual patient tolerance). By competitively binding to estrogen receptors or inhibiting estrogen production, hormonal therapies effectively disrupt the hormonal signaling pathways that fuel tumor growth. This method, aiming at the molecular underpinnings of breast cancer, underscores the significance of precise dosing and administration strategies to maximize therapeutic efficacy.

Combination therapy methods, integrating antineoplastic agents, are increasingly adopted to enhance treatment results (54). Antineoplastic combined chemotherapy protocols have proven to be particularly valuable in managing hormone receptor-positive breast cancers (55). The above-described protocols strategically incorporate chemotherapeutic agents alongside hormonal therapies, addressing the heterogeneity of cancer cells and mitigating the risk of resistance (56–58). Neoadjuvant therapy, a key context for the above-mentioned combination strategies, provides a unique opportunity to evaluate treatment response directly. This method enables clinicians to tailor subsequent therapeutic decisions, optimize surgical interventions, and refine treatment regimens based on individual patient needs.

However, the therapeutic benefits of antineoplastic agents (e.g., hormonal therapies) often come with potential adverse effects. Patients undergoing hormonal therapy may be subjected to a range of side effects (e.g., hot flashes, mood disturbances, and musculoskeletal symptoms) (59–62). To improve patient compliance and minimize discomfort, ongoing research is committed to finding innovative ways to mitigate the above-described adverse effects. Additionally, the evolution of antineoplastic agents continues, with investigations into novel agents and targeted therapies that offer the promise of expanding the treatment arsenal while striving to reduce side effects and enhance patient tolerability.

In brief, breast cancer therapy research has witnessed significant advancements in the use of antineoplastic agents, particularly hormonal therapies and combination protocols. The meticulous administration and dosing of the above-mentioned agents play a critical role in optimizing treatment results. As research and clinical practice continue to evolve, so too does our understanding of the complex interplay between therapy and adverse effects. This dynamic landscape underscores the significance of a multidisciplinary method, emphasizing individualized patient care and the ongoing exploration of innovative agents and methods to further enhance the efficacy and tolerability of breast cancer therapies.

#### 5.2.4 Estrogen receptors of breast cancer

Cluster 3 indicates a correlation with estrogen receptors of breast cancer. The role of estrogen receptors in breast cancer therapy research should be reasonably defined. Estrogen receptors take on critical significance in hormone receptor-positive breast cancers (63) that account for a significant proportion of cases (64). Insights should be gained into estrogen receptor metabolism and signaling pathways, which is crucial to the development of effective therapies. The above-described receptors are employed as vital biomarkers, which can guide treatment decisions and enable the tailoring of therapies to individual patients (65). Moreover, the analysis of estrogen receptor expression levels has become a standard practice, which can enable clinicians to divide patients in accordance with the likelihood of hormonal therapy response (66, 67). The evolving landscape of estrogen receptor research underscores the significance of continuous exploration into this critical aspect of breast cancer biology.

Breast cancer therapy research faces a challenge posed by the development of drug resistance, particularly in postmenopausal women (68, 69). Despite the initial success of hormonal therapies (e.g., Tamoxifen and Aromatase Inhibitors), resistance can be generated with time, which can limit the long-term efficacy of the above-mentioned treatments (70–72). Understanding how breast tumors adapt to evade hormonal therapies, including changes in estrogen and progesterone receptor expression, is essential for developing strategies to combat resistance. Ongoing research efforts are dedicated to identifying biomarkers and molecular pathways associated with drug resistance (73), with the ultimate goal of enhancing treatment results and prolonging the effectiveness of hormonal therapies in the postmenopausal population.

Hormone receptor analysis, which covers estrogen and progesterone receptors, can be recognized as a critical component of breast cancer therapy research. This analysis informs treatment decisions while deepening our understanding of the heterogeneous nature of breast cancer. Progesterone receptors (e.g., estrogen receptors) pronouncedly affect tumor biology and therapeutic responses (74, 75). The study of the above-described receptors provides insights into the complexities of hormone receptor-positive breast cancers and offers opportunities for more tailored therapies. In brief, hormone receptor analysis remains a linchpin in breast cancer therapy research, guiding clinical practice and driving the development of innovative treatment strategies aimed at improving patient results.

#### 5.2.5 Adherence and survival rates of breast cancer

Cluster 4 indicates a correlation with adherence and survival rates of breast cancer. Medication adherence has been considered a vital factor in the effectiveness of breast cancer therapy (76–78), and its implications are underscored by the statistical and numerical data at hand. Maintaining consistent and appropriate medication adherence is essential for achieving optimal treatment results. However, articles have consistently shown suboptimal adherence rates among breast cancer patients. The above-mentioned statistics highlight a critical area for improvement in breast cancer care. Addressing the factors contributing to non-adherence, such as medication side effects and psychological factors, is essential (79, 80). Strategies aimed at enhancing patient education and support (81), combined with innovative interventions like mobile health applications (82, 83), have the potential to improve medication adherence rates and, consequently, patient survival and quality of life.

Breast cancer still arouses major global health concern, and it exerts a huge epidemiological effect. The prevalence of breast cancer is underscored by the epidemiological data, and this data reveals that effective therapies and intervention strategies are urgently required (84). Despite advancements in early detection and treatment, breast cancer continues to be a leading cause of cancer-related mortality among women worldwide (85, 86). The epidemiological perspective serves as a reminder of the substantial burden that breast cancer places on healthcare systems and societies. It reinforces the significance of continued research into novel therapies, personalized treatment methods, and the psychological aspects of breast cancer care to reduce mortality rates and improve the overall well-being of breast cancer patients.

The psychological aspect of breast cancer, including the emotional and psychological responses of patients, is a significant factor to consider in the context of therapy research and mortality. Breast cancer diagnosis and treatment can evoke profound psychological distress, affecting patients’ mental health and quality of life (87, 88). Addressing the psychological effect exerted by breast cancer is not only critical to the well-being of patients, and it may also indirectly affect treatment results. Psychological support and interventions (e.g., counseling and support groups) can be crucial to mitigating the psychological toll of breast cancer, which may lead to enhanced medication adherence, treatment response, and overall survival rates (89–92). Recognizing the psychological dimension of breast cancer in the discussion of therapy research is vital for comprehensively addressing the complexities of this disease and its effect on patient mortality.

### 5.3 Limitation

The application of bibliometric assessment for research analysis is constrained by specific constraints. One limitation involves the potential omission of high-quality research from the dataset due to insufficient citation frequency. This circumstance can arise from the delayed recognition of recently published articles, as bibliometrics primarily relies on scrutinizing publication citation counts (93). Furthermore, the biclustering analysis employed to categorize and pinpoint prominent keywords might not encompass all pertinent subjects in a field of study. Additionally, the data acquired from the Web of Science database might lack the most current publications due to lag times in updates. Notwithstanding the above-described restrictions, bibliometric analysis remains a valuable instrument for obtaining a comprehensive and effective comprehension of the research landscape in a specific domain.

## 6 Conclusions

The field of breast cancer endocrine therapy has aroused rising attention over the past few years, such that the current research trends should be analyzed. This study provides a summary of key information regarding breast cancer endocrine therapy publications (e.g., the number of articles, country of origin, institutions involved, and the journals for publication). The results indicate that the US has made the most significant contribution to breast cancer endocrine therapy research. While challenges of drug resistance, medication adherence, and psychological support remain significant areas of concern, breast cancer endocrine therapy research has become the cornerstone of hormone receptor-positive breast cancer management. With the deepening of the understanding of estrogen and progesterone receptor signaling, coupled with the development of targeted interventions (e.g., Tamoxifen, aromatase inhibitors, and emerging agents), the significance of personalized treatment methods is increasingly emphasized. The above-mentioned therapies increase disease-free survival rates while contributing to the improvement of the quality of life for breast cancer patients. Further interdisciplinary collaboration, innovative interventions, and a deeper exploration of biomarkers and genetic factors will be instrumental in addressing the above-described challenges and refining the landscape of breast cancer endocrine therapy research to ultimately benefit patients worldwide. Hopefully, this study has the potential to offer valuable guidance for researchers worldwide.

## Data availability statement

The original contributions presented in the study are included in the article/supplementary material. Further inquiries can be directed to the corresponding author.

## Author contributions

DW: Conceptualization, Writing – original draft. YY: Formal analysis, Software, Writing – original draft. LY: Software, Writing – original draft. HY: Conceptualization, Writing – review & editing.

## References

[1] Cruceriu D Baldasici O Balacescu O Berindan-Neagoe I. The dual role of tumor necrosis factor-alpha (TNF-alpha) in breast cancer: molecular insights and therapeutic approaches. Cell Oncol (2020) 43:1–18. doi: 10.1007/s13402-019-00489-1 PMC12990688 31900901

[2] Agostinetto E Jacobs F Debien V De Caluwe A Pop CF Catteau X. Post-neoadjuvant treatment strategies for patients with early breast cancer. Cancers (2022) 14:5467. doi: 10.3390/cancers14215467 36358886 PMC9654353

[3] Sartaj A Qamar Z Qizilbash FF Md S Alhakamy NA Baboota S. Polymeric nanoparticles: exploring the current drug development and therapeutic insight of breast cancer treatment and recommendations. Polymers (2021) 13:4400. doi: 10.3390/polym13244400 34960948 PMC8703470

[4] Vagia ELN Cristofanilli MSM. New treatment strategies for the inflammatory breast cancer. Curr Treat Options In Oncol (2021) 22:50. doi: 10.1007/s11864-021-00843-2 33893888

[5] Zelnak AB O'Regan RM. Optimizing endocrine therapy for breast cancer. J Of Natl Compr Cancer Network. (2015) 13:E56–64. doi: 10.6004/jnccn.2015.0125 26285250

[6] Cristofanilli M Turner NC Bondarenko I Ro J Im SA Masuda N. Fulvestrant plus palbociclib versus fulvestrant plus placebo for treatment of hormone-receptor-positive, HER2-negative metastatic breast cancer that progressed on previous endocrine therapy (PALOMA-3): final analysis of the multicentre, double-blind, phase 3 randomised controlled trial. Lancet Oncol (2016) 17:425–39. doi: 10.1016/S1470-2045(15)00613-0 26947331

[7] Gradishar W. Taxanes for the treatment of metastatic breast cancer. Breast Cancer: Basic Clin Res (2012) 6. doi: 10.4137/BCBCR.S8205 PMC3486789 23133315

[8] Gil EMC. Targeting the PI3K/AKT/mTOR pathway in estrogen receptor-positive breast cancer. Cancer Treat Rev (2014) 40:862–71. doi: 10.1016/j.ctrv.2014.03.004 24774538

[9] Burstein HJ. Systemic therapy for estrogen receptor-positive, HER2-negative breast cancer. New Engl J Of Med (2020) 383:2557–70. doi: 10.1056/NEJMra1307118 33369357

[10] Joshi PA Jackson HW Beristain AG Di Grappa MA Mote PA Clarke CL. Progesterone induces adult mammary stem cell expansion. Nature. (2010) 465:803–7. doi: 10.1038/nature09091 20445538

[11] Russo J Russo IH. The role of estrogen in the initiation of breast cancer. J Of Steroid Biochem Mol Biol (2006) 102:89–96. doi: 10.1016/j.jsbmb.2006.09.004 17113977 PMC1832080

[12] Lowery AJ Miller N Devaney A McNeill RE Davoren PA Lemetre C. MicroRNA signatures predict oestrogen receptor, progesterone receptor and HER2/neu receptor status in breast cancer. Breast Cancer Res (2009) 11:R27. doi: 10.1186/bcr2257 19432961 PMC2716495

[13] Mohammed H Russell IA Stark R Rueda OM Hickey TE Tarulli GA. Progesterone receptor modulates ER alpha action in breast cancer. Nature. (2015) 523:313. doi: 10.1038/nature14583 26153859 PMC4650274

[14] Kulkoyluoglu E Madak-Erdogan Z. Nuclear and extranuclear-initiated estrogen receptor signaling crosstalk and endocrine resistance in breast cancer. Steroids. (2016) 114:41–7. doi: 10.1016/j.steroids.2016.06.007 27394959

[15] Kulkoyluoglu-Cotul E Arca A Madak-Erdogan Z. Crosstalk between estrogen signaling and breast cancer metabolism. Trends In Endocrinol Metab (2019) 30:25–38. doi: 10.1016/j.tem.2018.10.006 30471920

[16] Pagliuca M Donato M D’Amato AL Rosanova M Russo AOM Scafetta R. New steps on an old path: Novel estrogen receptor inhibitors in breast cancer. Crit Rev Oncology/Hematology. (2022) 180:103861. doi: 10.1016/j.critrevonc.2022.103861 36374739

[17] Fisher B Costantino J Redmond C Poisson R Bowman D Couture J. A randomized clinical trial evaluating tamoxifen in the treatment of patients with node-negative breast cancer who have estrogen-receptor-positive tumors. New Engl J Of Med (1989) 320:479–84. doi: 10.1056/NEJM198902233200802 2644532

[18] Smith IE Dowsett M. Aromatase inhibitors in breast cancer. New Engl J Of Med (2003) 348:2431–42. doi: 10.1056/NEJMra023246 12802030

[19] Gao CD Li HY Zhou C Liu C Zhuang J Liu LJ. Survival-associated metabolic genes and risk scoring system in HER2-positive breast cancer. Front In Endocrinol (2022) 13. doi: 10.3389/fendo.2022.813306 PMC9161264 35663326

[20] Manohar PM Davidson NE. Updates in endocrine therapy for metastatic breast cancer. Cancer Biol Med (2022) 19:202–12. doi: 10.20892/j.issn.2095-3941.2021.0255 PMC8832960 34609096

[21] Finitsis DJ Vose BA Mahalak JG Salner AL. Interventions to promote adherence to endocrine therapy among breast cancer survivors: A meta-analysis. Psycho-Oncology. (2019) 28:255–63. doi: 10.1002/pon.4959 30511789

[22] Punglia RS Kuntz KM Winer EP Weeks JC Burstein HJ. Optimizing adjuvant endocrine therapy in postmenopausal women with early-stage breast cancer: A decision analysis. J Of Clin Oncol (2005) 23:5178–87. doi: 10.1200/JCO.2005.02.964 15998905

[23] Harbeck N Rastogi P Martin M Tolaney SM Shao ZM Fasching PA. Adjuvant abemaciclib combined with endocrine therapy for high-risk early breast cancer: updated efficacy and Ki-67 analysis from the monarchE study. Ann Of Oncol (2021) 32:1571–81. doi: 10.1016/j.annonc.2021.09.015 34656740

[24] Kim HSJ Wahid M Choi C Das P Jung S Khosa F. Bibliometric analysis of manuscript characteristics that influence citations: A comparison of ten major dermatology journals. Burns. (2020) 46:1686–92. doi: 10.1016/j.burns.2020.05.002 32536449

[25] Chen X Xiao H Zhao Q Xu X Cen Y Xiao D. Research hotspot and trend of microneedles in biomedical field: A bibliometric analysis from 2011 to 2020. Burns. (2022) 48:959–72. doi: 10.1016/j.burns.2022.04.004 35504768

[26] Chen X Shi X Xiao H Xiao D Xu X. Research hotspot and trend of chronic wounds: A bibliometric analysis from 2013 to 2022. Wound Repair Regen (2023) 31:597–612. doi: 10.1111/wrr.13117 37552080

[27] Harris TJR McCormick F. The molecular pathology of cancer. Nat Rev Clin Oncol (2010) 7:251–65. doi: 10.1038/nrclinonc.2010.41 20351699

[28] Bartlett JMS Brookes CL Robson T van de Velde CJH Billingham LJ Campbell FM. Estrogen receptor and progesterone receptor as predictive biomarkers of response to endocrine therapy: A prospectively powered pathology study in the tamoxifen and exemestane adjuvant multinational trial. J Of Clin Oncol (2011) 29:1531–8. doi: 10.1200/JCO.2010.30.3677 PMC3082973 21422407

[29] Wei S Kozono S Kats L Nechama M Li WZ Guarnerio J. Active Pin1 is a key target of all-trans retinoic acid in acute promyelocytic leukemia and breast cancer. Nat Med (2015) 21:457–U230. doi: 10.1038/nm.3839 25849135 PMC4425616

[30] Nedeljkovic M Damjanovic A. Mechanisms of chemotherapy resistance in triple-negative breast cancer-how we can rise to the challenge. Cells. (2019) 8:957. doi: 10.3390/cells8090957 31443516 PMC6770896

[31] Zhang L Zhou FF de Vinuesa AG de Kruijf EM Mesker WE Hui L. TRAF4 promotes TGF-beta receptor signaling and drives breast cancer metastasis. Mol Cell (2013) 51:559–72. doi: 10.1016/j.molcel.2013.07.014 23973329

[32] Goutsouliak K Veeraraghavan J Sethunath V De Angelis C Osborne CK Rimawi MF. Towards personalized treatment for early stage HER2-positive breast cancer. Nat Rev Clin Oncol (2020) 17:233–50. doi: 10.1038/s41571-019-0299-9 PMC8023395 31836877

[33] Chan CWH Law BMH So WKW Chow KM Waye MMY. Novel strategies on personalized medicine for breast cancer treatment: an update. Int J Of Mol Sci (2017) 18:2423. doi: 10.3390/ijms18112423 29140300 PMC5713391

[34] Xiao Y Ma D Yang YS Yang F Ding JH Gong Y. Comprehensive metabolomics expands precision medicine for triple-negative breast cancer. Cell Res (2022) 32:477–90. doi: 10.1038/s41422-022-00614-0 PMC9061756 35105939

[35] Yan YY He M Zhao L Wu HZ Zhao YY Han L. A novel HIF-2 alpha targeted inhibitor suppresses hypoxia-induced breast cancer stemness via SOD2-mtROS-PDI/GPR78-UPRER axis. Cell Death Differentiation. (2022) 29:1769–89. doi: 10.1038/s41418-022-00963-8 PMC9433403 35301432

[36] Zhu WJ Chen X Guo XY Liu HT Ma RR Wang YW. Low glucose-induced overexpression of HOXC-AS3 promotes metabolic reprogramming of breast cancer. Cancer Res (2022) 82:805–18. doi: 10.1158/0008-5472.CAN-21-1179 35031573

[37] Wang TY Fahrmann JF Lee H Li YJ Tripathi SC Yue CY. JAK/STAT3-regulated fatty acid beta-oxidation is critical for breast cancer stem cell self-renewal and chemoresistance. Cell Metab (2018) 27:136. doi: 10.1016/j.cmet.2017.11.001 29249690 PMC5777338

[38] Varghese E Samuel SM Liskova A Samec M Kubatka P Busselberg D. Targeting glucose metabolism to overcome resistance to anticancer chemotherapy in breast cancer. Cancers. (2020) 12:2252. doi: 10.3390/cancers12082252 32806533 PMC7464784

[39] Forbes NS Meadows AL Clark DS Blanch HW. Estradiol stimulates the biosynthetic pathways of breast cancer cells: Detection by metabolic flux analysis. Metab Engineering. (2006) 8:639–52. doi: 10.1016/j.ymben.2006.06.005 16904360

[40] Lenz G Hamilton A Geng SH Hong T Kalkum M Momand J. t-darpp activates IGF-1R signaling to regulate glucose metabolism in trastuzumab-resistant breast cancer cells. Clin Cancer Res (2018) 24:1216–26. doi: 10.1158/1078-0432.CCR-17-0824 PMC7007690 29180608

[41] Iqbal MA Siddiqui S Rehman AU Siddiqui FA Singh P Kumar B. Multiomics integrative analysis reveals antagonistic roles of CBX2 and CBX7 in metabolic reprogramming of breast cancer. Mol Oncol (2021) 15:1450–65. doi: 10.1002/1878-0261.12894 PMC8096797 33400401

[42] Li GQ Xie SJ Wu SG He ZY. Impact of the 21-gene expression assay on treatment decisions and clinical outcomes in breast cancer with one to three positive lymph nodes. Front In Endocrinology. (2023) 14. doi: 10.3389/fendo.2023.1103949 PMC9980792 36875478

[43] Yan H Shang WT Sun XD Zhao LY Wang XM Zhang SX. Neoadjuvant nano-photothermal therapy used before operation effectively assists in surgery for breast cancer. Nanoscale. (2019) 11:706–16. doi: 10.1039/C8NR08109C 30565621

[44] Ilgun AS Aktepe F Gonullu O Kapucuoglu N Yararbas K Alco G. The effect of neoadjuvant chemotherapy on tumor-infiltrating lymphocytes in patients with breast cancer. Future Oncol (2022) 18:3289–98. doi: 10.2217/fon-2022-0157 36017739

[45] Nikolai BC Lanz RB York B Dasgupta S Mitsiades N Creighton CJ. HER2 signaling drives DNA anabolism and proliferation through SRC-3 phosphorylation and E2F1-regulated genes. Cancer Res (2016) 76:1463–75. doi: 10.1158/0008-5472.CAN-15-2383 PMC4794399 26833126

[46] Albain K Anderson S Arriagada R Barlow W Bergh J Bliss J. Comparisons between different polychemotherapy regimens for early breast cancer: meta-analyses of long-term outcome among 100 000 women in 123 randomised trials. Lancet. (2012) 379:432–44. doi: 10.1016/S0140-6736(11)61625-5 PMC3273723 22152853

[47] Bowers LW Maximo IXF Brenner AJ Beeram M Hursting SD Price RS. NSAID use reduces breast cancer recurrence in overweight and obese women: role of prostaglandin-aromatase interactions. Cancer Res (2014) 74:4446–57. doi: 10.1158/0008-5472.CAN-13-3603 PMC12935481 25125682

[48] Osborne CK Neven P Dirix LY Mackey JR Robert J Underhill C. Gefitinib or placebo in combination with tamoxifen in patients with hormone receptor-positive metastatic breast cancer: A randomized phase II study. Clin Cancer Res (2011) 17:1147–59. doi: 10.1158/1078-0432.CCR-10-1869 PMC3074404 21220480

[49] Morandi P Rouzier R Altundag K Buzdar AU Theriault RL Hortobagyi G. The role of aromatase inhibitors on the adjuvant treatment of breast carcinoma - The M. D. Anderson cancer center evidence-based approach. Cancer. (2004) 101:1482–9. doi: 10.1002/cncr.20522 15378476

[50] Colozza M Califano R Minenza E Dinh P Azambuja E. Aromatase inhibitors: A new reality for the adjuvant endocrine treatment of early-stage breast cancer in postmenopausal women. Mini-Reviews In Medicinal Chem (2008) 8:564–74. doi: 10.2174/138955708784534472 18537711

[51] Boraei ATA Singh PK Sechi M Satta S. Discovery of novel functionalized 1,2,4-triazoles as PARP-1 inhibitors in breast cancer: Design, synthesis and antitumor activity evaluation. Eur J Of Medicinal Chem (2019) 182:111621. doi: 10.1016/j.ejmech.2019.111621 31442685

[52] Farghaly TA Abbas IM Hassan WMI Lotfy MS Al-Qurashi NT Ben Hadda T. Structure determination and quantum chemical analysis of 1,3-dipolar cycloaddition of nitrile imines and new dipolarophiles and POM analyses of the products as potential breast cancer inhibitors. Russian J Of Organic Chem (2020) 56:1258–71. doi: 10.1134/S1070428020070210

[53] Howell A Cuzick J Baum M Buzdar A Dowsett M Forbes JF. Results of the ATAC (Arimidex, Tamoxifen, Alone or in Combination) trial after completion of 5 years' adjuvant treatment for breast cancer. Lancet. (2005) 365:60–2. doi: 10.1016/S0140-6736(04)17666-6 15639680

[54] Li ZY Zheng JH Ji ZQ Chen LZ Wu JY Zou J. Addition of capecitabine to adjuvant chemotherapy may be the most effective strategy for patients with early-stage triple-negative breast cancer: A network meta-analysis of 9 randomized controlled trials. Front In Endocrinol (2022) 13. doi: 10.3389/fendo.2022.939048 PMC9358934 35957836

[55] Yaghi M Bilani N Dominguez B Jabbal IS Rivera C Zerdan MB. Management of HR+/HER2+lobular breast cancer and trends do not mirror better outcomes. Breast. (2022) 64:112–20. doi: 10.1016/j.breast.2022.05.005 PMC9157253 35640346

[56] Telli ML Carlson RW. First-line chemotherapy for metastatic breast cancer. Clin Breast Cancer. (2009) 9:S66–72. doi: 10.3816/CBC.2009.s.007 19596645

[57] Hurvitz S Mead M. Triple-negative breast cancer: advancements in characterization and treatment approach. Curr Opin Obstet Gynecol (2016) 28:59–69. doi: 10.1097/GCO.0000000000000239 26694831

[58] Abotaleb M Kubatka P Caprnda M Varghese E Zolakova B Zubor P. Chemotherapeutic agents for the treatment of metastatic breast cancer: An update. Biomedicine Pharmacotherapy. (2018) 101:458–77. doi: 10.1016/j.biopha.2018.02.108 29501768

[59] Mann E Smith MJ Hellier J Balabanovic JA Hamed H Grunfeld EA. Cognitive behavioural treatment for women who have menopausal symptoms after breast cancer treatment (MENOS 1): a randomised controlled trial. Lancet Oncol (2012) 13:309–18. doi: 10.1016/S1470-2045(11)70364-3 PMC3314999 22340966

[60] Markovitz LC Drysdale NJ Bettencourt BA. The relationship between risk factors and medication adherence among breast cancer survivors: What explanatory role might depression play? Psycho-Oncology (2017) 26:2294–9. doi: 10.1002/pon.4362 28032940

[61] Ugras SK Rahman RL. Hormone replacement therapy after breast cancer: Yes, No or maybe? Mol. Cell. Endocrinol. (2021) 525:111180. doi: 10.1016/j.mce.2021.111180 33508379

[62] Hershman DL Unger JM Greenlee H Capodice JL Lew DL Darke AK. Effect of acupuncture vs sham acupuncture or waitlist control on joint pain related to aromatase inhibitors among women with early-stage breast cancer A randomized clinical trial. Jama-Journal Of Am Med Assoc (2018) 320:167–76. doi: 10.1001/jama.2018.8907 PMC6583520 29998338

[63] Rani A Stebbing J Giamas G Murphy J. Endocrine resistance in hormone receptor positive breast cancer-from mechanism to therapy. Front In Endocrinology. (2019) 10. doi: 10.3389/fendo.2019.00245 PMC6543000 31178825

[64] Reinert T Cascelli F De Resende CAA Goncalves AC Godo VSP Barrios CH. Clinical implication of low estrogen receptor (ER-low) expression in breast cancer. Front In Endocrinology. (2022) 13. doi: 10.3389/fendo.2022.1015388 PMC9729538 36506043

[65] Xia YL He XP Renshaw L Martinez-Perez C Kay C Gray M. Integrated DNA and RNA sequencing reveals drivers of endocrine resistance in estrogen receptor-positive breast cancer. Clin Cancer Res (2022) 28:3618–29. doi: 10.1158/1078-0432.CCR-21-3189 PMC7613305 35653148

[66] Alfarsi LH El Ansari R Craze ML Toss MS Masisi B Ellis IO. CDC20 expression in oestrogen receptor positive breast cancer predicts poor prognosis and lack of response to endocrine therapy. Breast Cancer Res And Treat (2019) 178:535–44. doi: 10.1007/s10549-019-05420-8 31471836

[67] Madeira M Mattar A Logullo AF Soares FA Gebrim LH. Estrogen receptor alpha/beta ratio and estrogen receptor beta as predictors of endocrine therapy responsiveness-a randomized neoadjuvant trial comparison between anastrozole and tamoxifen for the treatment of postmenopausal breast cancer. BMC Cancer. (2013) 13:425. doi: 10.1186/1471-2407-13-425 24047421 PMC3851532

[68] De Amicis F Thirugnansampanthan J Cui YK Selever J Beyer A Parra I. Androgen receptor overexpression induces tamoxifen resistance in human breast cancer cells. Breast Cancer Res And Treat (2010) 121:1–11. doi: 10.1007/s10549-009-0436-8 19533338 PMC2995248

[69] Wegman P Elingarami S Carstensen J Stal O Nordenskjold B Wingren S. Genetic variants of CYP3A5, CYP2D6, SULT1A1, UGT2B15 and tamoxifen response in postmenopausal patients with breast cancer. Breast Cancer Res (2007) 9:R7. doi: 10.1186/bcr1640 17244352 PMC1851378

[70] Osipo C Gajdos C Cheng D Jordan VC. Reversal of tamoxifen resistant breast cancer by low dose estrogen therapy. J Of Steroid Biochem And Mol Biol (2005) 93:249–56. doi: 10.1016/j.jsbmb.2004.12.005 15860267

[71] Martin M Zielinski C Ruiz-Borrego M Carrasco E Turner N Ciruelos EM. Palbociclib in combination with endocrine therapy versus capecitabine in hormonal receptor-positive, human epidermal growth factor 2-negative, aromatase inhibitor-resistant metastatic breast cancer: a phase III randomised controlled trial-PEARL. Ann Of Oncol (2021) 32:488–99. doi: 10.1016/j.annonc.2020.12.013 33385521

[72] Maximov PY Fan P Abderrahman B Curpan R Jordan VC. Estrogen receptor complex to trigger or delay estrogen-induced apoptosis in long-term estrogen deprived breast cancer. Front In Endocrinology. (2022) 13. doi: 10.3389/fendo.2022.869562 PMC8960923 35360069

[73] Haque MM Desai KV. Pathways to endocrine therapy resistance in breast cancer. Front In Endocrinology. (2019) 10. doi: 10.3389/fendo.2019.00573 PMC6712962 31496995

[74] Singhal H Greene ME Tarulli G Zarnke AL Bourgo RJ Laine M. Genomic agonism and phenotypic antagonism between estrogen and progesterone receptors in breast cancer. Sci Adv (2016) 2:e1501924. doi: 10.1126/sciadv.1501924 27386569 PMC4928895

[75] Knutson TP Truong TH Ma S Brady NJ Sullivan ME Raj G. Posttranslationally modified progesterone receptors direct ligand-specific expression of breast cancer stem cell-associated gene programs. J Of Hematol Oncol (2017) 10:89. doi: 10.1186/s13045-017-0462-7 28412963 PMC5392969

[76] Lambert LK Balneaves LG Howard AF Chia SLK Gotay CC. Healthcare provider perspectives on adherence to adjuvant endocrine therapy after breast cancer. Curr Oncol (2021) 28:1472–82. doi: 10.3390/curroncol28020139 PMC8167827 33918560

[77] Bright EE Stanton AL. Prospective investigation of social support, coping, and depressive symptoms: A model of adherence to endocrine therapy among women with breast cancer. J Of Consulting And Clin Psychol (2018) 86:242–53. doi: 10.1037/ccp0000272 29265835

[78] Smith SG Sestak I Forster A Partridge A Side L Wolf MS. Factors affecting uptake and adherence to breast cancer chemoprevention: a systematic review and meta-analysis. Ann Of Oncol (2016) 27:575–90. doi: 10.1093/annonc/mdv590 PMC4803450 26646754

[79] Nestoriuc Y von Blanckenburg P Schuricht F Barsky AJ Hadji P Albert US. Is it best to expect the worst? Influence of patients' side-effect expectations on endocrine treatment outcome in a 2-year prospective clinical cohort study. Ann Of Oncol (2016) 27:1909–15. doi: 10.1093/annonc/mdw266 27551051

[80] von Blanckenburg P Schuricht F Albert US Rief W Nestoriuc Y. Optimizing expectations to prevent side effects and enhance quality of life in breast cancer patients undergoing endocrine therapy: study protocol of a randomized controlled trial. BMC Cancer. (2013) 13:426. doi: 10.1186/1471-2407-13-426 24047450 PMC3848828

[81] Friese CR Pini TM Li Y Abrahamse PH Graff JJ Hamilton AS. Adjuvant endocrine therapy initiation and persistence in a diverse sample of patients with breast cancer. Breast Cancer Res And Treat (2013) 138:931–9. doi: 10.1007/s10549-013-2499-9 PMC3633703 23542957

[82] Lozano-Lozano M Martin-Martin L Galiano-Castillo N Alvarez-Salvago F Cantarero-Villanueva I Fernandez-Lao C. Integral strategy to supportive care in breast cancer survivors through occupational therapy and a m-health system: design of a randomized clinical trial. BMC Med Inf And Decision Making (2016) 16:150. doi: 10.1186/s12911-016-0394-0 PMC5124301 27887610

[83] Handa S Okuyama H Yamamoto H Nakamura S Kato Y. Effectiveness of a smartphone application as a support tool for patients undergoing breast cancer chemotherapy: A randomized controlled trial. Clin Breast Cancer. (2020) 20:201–8. doi: 10.1016/j.clbc.2020.01.004 32201165

[84] Ginsberg GM Lauer JA Zelle S Baeten S Baltussen R. Cost effectiveness of strategies to combat breast, cervical, and colorectal cancer in sub-Saharan Africa and South East Asia: mathematical modelling study. Bmj-British Med J (2012) 344:e614. doi: 10.1136/bmj.e614 PMC3292522 22389347

[85] Basu P Maier C. Phytoestrogens and breast cancer: *In vitro* anticancer activities of isoflavones, lignans, coumestans, stilbenes and their analogs and derivatives. Biomedicine Pharmacotherapy. (2018) 107:1648–66. doi: 10.1016/j.biopha.2018.08.100 30257383

[86] DeSantis CE Bray F Ferlay J Lortet-Tieulent J Anderson BO Jemal A. International variation in female breast cancer incidence and mortality rates. Cancer Epidemiol Biomarkers Prev (2015) 24:1495–506. doi: 10.1158/1055-9965.EPI-15-0535 26359465

[87] Perez-Tejada J Labaka A Pascual-Sagastizabal E Garmendia L Iruretagoyena A Arregi A. Predictors of psychological distress in breast cancer survivors: A biopsychosocial approach. Eur J Of Cancer Care (2019) 28:e13166. doi: 10.1111/ecc.13166 31571327

[88] Fortin J Leblanc M Elgbeili G Cordova MJ Marin MF Brunet A. The mental health impacts of receiving a breast cancer diagnosis: A meta-analysis. Br J Of Cancer. (2021) 125:1582–92. doi: 10.1038/s41416-021-01542-3 PMC8608836 34482373

[89] Benedict C Thom B Teplinsky E Carleton J Kelvin JF. Family-building after breast cancer: considering the effect on adherence to adjuvant endocrine therapy. Clin Breast Cancer. (2017) 17:165–70. doi: 10.1016/j.clbc.2016.12.002 28087390

[90] van de Wiel HJ Stuiver MM May AM van Grinsven S Aaronson NK Oldenburg HSA. Effects of and lessons learned from an internet-based physical activity support program (with and without physiotherapist telephone counselling) on physical activity levels of breast and prostate cancer survivors: the PABLO randomized controlled trial. Cancers (2021) 13:3665. doi: 10.3390/cancers13153665 34359567 PMC8345041

[91] Esplen MJ Leszcz M Hunter J Wong J Leung YW Toner B. A randomized controlled trial of a supportive expressive group intervention for women with a family history of breast cancer. Psycho-Oncology (2018) 27:2645–53. doi: 10.1002/pon.4822 29952047

[92] Rutherford CL Goodman D Lannigan A. A systematic literature review of the management, oncological outcomes and psychosocial implications of male breast cancer. EJSO (2022) 48:2104–11. doi: 10.1016/j.ejso.2022.06.004 35725681

[93] Wallin JA. Bibliometric methods: pitfalls and possibilities. Basic Clin Pharmacol toxicology. (2005) 97:261–75. doi: 10.1111/j.1742-7843.2005.pto_139.x 16236137

